# Nematocidal and Bactericidal Activities of Green Synthesized Silver Nanoparticles Mediated by *Ficus sycomorus* Leaf Extract

**DOI:** 10.3390/life13051083

**Published:** 2023-04-25

**Authors:** Dina Elkobrosy, Abdulaziz A. Al-Askar, Hamada El-Gendi, Yiming Su, Rokaia Nabil, Ahmed Abdelkhalek, Said Behiry

**Affiliations:** 1 Plant Protection and Biomolecular Diagnosis Department, Arid Lands Cultivation Research Institute, City of Scientific Research and Technological Applications (SRTA-City), Alexandria 21934, Egypt; 2 Department of Botany and Microbiology, College of Science, King Saud University, P.O. Box 2455, Riyadh 11451, Saudi Arabia; 3 Bioprocess Development Department, Genetic Engineering and Biotechnology Research Institute, City of Scientific Research and Technological Applications, New Borg El-Arab City 21934, Egypt; 4 Utah Water Research Laboratory, Department of Civil and Environmental Engineering, Utah State University, Logan, UT 84341, USA; 5 Agricultural Botany Department, Faculty of Agriculture (Saba Basha), Alexandria University, Alexandria 21531, Egypt

**Keywords:** *Meloidogyne incognita*, silver nanoparticles, green synthesis, *Pectobacterium carotovorum*, antimicrobial property, plant pathogens, nematocidal activity, *Ficus sycomorus*, FTIR, SEM

## Abstract

Nanoparticles effectively control most plant pathogens, although research has focused more on their antimicrobial than their nematocidal properties. This study synthesized silver nanoparticles (Ag-NPs) through a green biosynthesis method using an aqueous extract of *Ficus sycomorus* leaves (FS-Ag-NPs). The nanoparticles were characterized using SEM, TEM, EDX, zeta sizer, and FTIR. The TEM results showed that the synthesized NPs were nanoscale and had an average particle size of 33 ± 1 nm. The elemental silver signal at 3 keV confirmed the formation of Ag-NPs from an aqueous leaf extract of *F. sycomorus*. The FTIR analysis revealed the existence of several functional groups in the prepared Ag-NPs. The strong-broad band detected at 3430 cm^−1^ indicated the stretching vibration of -OH (hydroxyl) and -NH_2_ (amine) groups. The nematocidal activity of biosynthesized FS-Ag-NPs has been evaluated in vitro against the root-knot nematode *Meloidogyne incognita* at 24, 48, and 72 h. The FS-Ag-NPs at a 200 µg/mL concentration applied for 48 h showed the highest effectiveness, with 57.62% nematode mortality. Moreover, the biosynthesized FS-Ag-NPs were also tested for their antibacterial activity against *Pectobacterium carotovorum*, *P. atrosepticum*, and *Ralstonia solanacearum*. With the application of nanoparticles, the reduction in bacterial growth gradually increased. The most potent activity at all concentrations was found in *R. solanacearum*, with values of 14.00 ± 2.16, 17.33 ± 2.05, 19.00 ± 1.41, 24.00 ± 1.41, and 26.00 ± 2.83 at concentrations of 5, 10, 15, 20, and 25 µg/mL, respectively, when compared with the positive control (Amoxicillin 25 µg) with a value of 16.33 ± 0.94. At the same time, the nanoparticles showed the lowest reduction values against *P. atrosepticum* when compared to the control. This study is the first report on the nematocidal activity of Ag-NPs using *F. sycomorus* aqueous extract, which could be a recommended treatment for managing plant-parasitic nematodes due to its simplicity, stability, cost-effectiveness, and environmentally safe nature.

## 1. Introduction

Nanotechnology is being used in other areas of science and technology to produce new nanoscale materials, pushing this field of study to the front of the scientific community [1]. Many of the physical and chemical properties of nanoparticles can be controlled by choosing their size and shape. Since nanoparticles (NPs) could be used in optoelectronics, recording media, sensing devices, catalysis, and medicine, producing and studying them is an important area of research [2]. Chemical methods used to produce nanoparticles today cannot be used in biomedicine because they use toxic chemicals and produce harmful byproducts. On the other hand, physical methods of producing nanoparticles, such as sputtering deposition, thin films, etc., were known to be difficult to use. As such, it is important to find new ways to produce nanoparticles that are safe for people to use, will not hurt the environment, will not break the bank, and will not kill animals [3]. Instead of using dangerous chemicals or dangerous processes to produce nanomaterials, biological methods can be used instead. Biomimetic systems include the use of yeast, fungi, bacteria, plants, and other organisms to produce nanostructures of metals and semiconductors that are safe for living things. Silver nanoparticles (Ag-NPs) are becoming more popular as more pathogens become resistant to antibiotics [2,4]. As a possible antimicrobial agent, metallic silver in the form of nanoparticles has received significant attention [5]. The inclusion of silver has been shown to be an effective way to slow down or stop microbial infections. Silver is also known to have an oligodynamic effect because it can prevent infections at very low concentrations [6].

*Ficus sycomorus*, a member of the Moraceae family, has been found to possess properties that inhibit the growth of bacteria [7,8]. Additionally, the aqueous extract of the plant contains several active components, including saponins, phenolic compounds, tannins, flavonoids, and alkaloids, all of which have pharmacological effects [9].

Plant-parasitic nematodes are a major threat to agricultural production because they are soil-borne diseases that infect the roots of most crops, thereby lowering crop yield and quality [10]. Among them, root-knot nematodes, also known as *Meloidogyne incognita*, are a type of soil-borne disease that can infect nearly all types of cultivated plants worldwide, resulting in substantial economic losses [11,12]. Plant pathogenic bacteria are among the most significant plant pathogens and can be found all over the world [13]. It is estimated that out of 7100 identified bacteria, around 150 species are responsible for the various diseases that might affect plants [14]. Among them, *Pectobacterium carotovorum*, *Erwinia amylovora*, and *Ralstonia solanacearum* are harmful plant pathogens that cause diseases called bacterial soft rot, fire blight, and brown rot, respectively. It can live on many different kinds of plants, some of which are important crops, including most vegetables and ornamental plants [15,16]. Bacterial pectolytic enzymes break down the pectin-rich middle lamella that holds plant cells together. This causes the cells to separate and damages the plant. The disease can be spread by water, insects, or tools such as sickles [13,15,16]. Several studies have been conducted on the effects of green-made Ag-NPs produced from different extracts on plant pathogens. In one such study, it was shown that *E. amylovora*, *P. carotovorum*, *R. solanacearum*, and *Xanthomonas citri* could not grow as a result of Ag-NPs produced from quercus [17]. *Erwinia cacticida* and *Citrobacter freundii* could not grow when Ag-NPs produced from *Piper nigrum* leaf extract were added [18].

Recently, it was found that Ag-NPs produced from the supernatant of the bacterium *Pseudomonas rhodesiae* were antibacterial against the soft rot pathogen *Dickeya dadantii* [19]. Pandey et al. [20] reviewed the benefits of Ag-NPs for crop protection and found that they can reduce inoculum accumulation, inhibit pathogen growth, lessen the severity of disease, lessen post-harvest losses, offer protection from disease, be effective at low doses, induce resistance, and boost yields. Dzimitrowicz et al. [21] and Spagnoletti et al. [22] are two of the few studies that examined the effectiveness of green or chemically produced Ag-NPs against the pathogen *P. carotovorum*. The current study aims to use an aqueous extract of *Ficus sycomorus* leaves as a stabilizing agent to achieve the green biosynthesis of Ag-NPs. The biosynthesized Ag-NPs were further investigated using several analytical techniques, including scanning electron microscopy (SEM), transmission electron microscopy (TEM), Fourier-transform infrared spectroscopy (FTIR), energy dispersive X-ray spectroscopy (EDX), and zeta potential. The nematocidal efficacy of biosynthesized Ag-NPs in inhibiting nematode activity (mortality%) as well as bactericidal activity against three plant pathogenic bacteria were evaluated. The nematocidal activity of green synthesized Ag-NPs from *F. sycomorus* aqueous extract against plant root-knot nematode was investigated for the first time in this study.

## 2. Materials and Methods

### 2.1. Preparation of Ag-NPs through Ficus sycomorus Leaves Extract

*Ficus sycomorus* leaf extract was used for the green synthesis of silver nanoparticles (Ag-NPS) using AgNO_3_ (Sigma Aldrich, St. Louis, MO, USA) as the starting precursor (our new reference). First, the *F. sycomorus* leaves were collected, rinsed thoroughly with tap water, and left to air dry. Then, the leaves were powdered using a mortar and pestle and sieved with a 100-mesh sieve (Advantech Manufacturing, Inc., New Berlin, WI, USA). The *F. sycomorus* extract was freshly prepared by mixing 10 g of *F. sycomorus* leaf powder with 100 mL of double distilled water (ddW) and shaking for 2 h at 50 °C in a shaking water bath (Fisherbrand™ Isotemp™ Shaking Water Bath, Thermo Fisher Scientific Inc., Waltham, MA, USA). Then, the extract was filtered with Whatman filter paper no.1. Afterward, 10 mL of clear extract filtrate was added to 90 mL of freshly prepared AgNO_3_ solution (1 mM). The solution was kept in dark conditions while being mixed. The reddish-brown color development indicated the formation of *F. sycomorus*-Ag-NPs (FS-Ag-NPs). At the end of the reaction, the resultant Ag-NPs were separated by centrifugation (6000 rpm) for 10 min in an Eppendorf Centrifuge 5810R (Eppendorf Corporate, Hamburg, Germany). The precipitated Ag-NPs was washed several times with ddW and dried overnight at 50 °C in a lab oven (Memmert GmbH + Co.KG, Schwabach, Germany).

### 2.2. Characterization of the Green Synthesized Ag-NPs

Different experimental approaches were used to characterize the green synthesized FS-Ag-NPs as follows: the surface-morphological structures of the produced FS-Ag-NPs were examined through scanning electron microscopy (SEM) using the JSM-6360 LA microscope (JEOL, Tokyo, Japan). The shape of the particles was determined through transmission electron microscopy (TEM, JSM-6360, JEOL, Tokyo, Japan), where the elemental analysis of FS-Ag-NPs was conducted through the energy-dispersive X-ray spectroscopy (EDX) unit combined with TEM. Furthermore, Fourier-transform infrared spectroscopy (FTIR, Agilent Technologies, Inc., Santa Clara, CA, USA) was used to evaluate the functional groups on the prepared FS-Ag-NPs using the KBr-disc approach. The zeta potential for prepared FS-Ag-NPs was elucidated through Zetasizer ver. 6.2 (ZS, Malvern, Germany).

### 2.3. The Origin of Root-Knot Nematode

Root-knot nematodes were isolated from pure cultures of *Meloidogyne incognita* preserved in a greenhouse on tomato plants (*Solanum lycopersicum*). *Meloidogyne incognita*-infected roots were collected and washed to remove the adhering soil particles, and eggs were isolated from them using the sodium hypochlorite method [23]. Second-stage juveniles (J2) were allowed to hatch for 48 h in a Petri dish filled with distilled water in an incubator at 27 °C.

### 2.4. Preparation of Silver Nanoparticles Concentrations and Their Effect on Nematodes

Using distilled sterile water, the FS-Ag-NPs were prepared in three concentrations (100, 200, and 400 µg/mL). One mL of root-knot nematode suspension was added to 1 mL of FS-Ag-NPs to obtain the final concentrations of FS-Ag-NPs (50, 100, and 200 µg/mL) in order to evaluate the viability of nematodes in vitro after 24, 48, and 72 h of exposure at room temperature. A nematode suspension without the addition of FS-Ag-NPs was used as a negative control. Each treatment was replicated thrice. The effect of FS-Ag-NPs on live nematodes, when compared with the control, was noticed and recorded using a stereomicroscope MZ16 A (Leica Microsystems GmbH, Wetzlar, Germany). In addition, the mortality percentage was counted.

### 2.5. Preparation of Bacterial Strains and Antimicrobial Activity Assay

*Pectobacterium carotovorum*, *P. atrosepticum*, and *Ralstonia solanacearum* bacterial isolates were isolated, identified previously, and accessioned with GenBank numbers MN598002, MG706146, and LN681200, respectively. All bacterial strains were grown in Luria broth (Merck, Germany) for 24 h at 30 °C and agitated at 200 rpm.

The disc diffusion method [24] was used to test the antibacterial properties of the synthesized silver nanoparticles. The overnight culture bacteria were adjusted in order to be taken and spread on agar plates to grow a uniform microbial growth plate. The tested concentrations of 5, 10, 15, 20, and 25 µg/mL were prepared from the FS-Ag-NPs, and each sterile disc received 15 µL of the used concentrations. As a control group for antibacterial activity, sterile double distilled water and the antibiotic amoxicillin (25 µg/disc) were used as negative and positive controls, respectively. Then, the discs were placed on the inoculated plates and kept at 30 °C for 24 h. The zone of inhibition (mm) diameter was measured and compared to the control groups. This experiment was carried out in triplicate.

### 2.6. Statistical Analysis

The biological experiments were performed three times. The results are given as the data average plus the standard deviation. Variance analysis was used to determine if the means of the data sets were the same. Tukey post-hoc-tests were perfoemed between the groups of interest and a *p*-value of less than 0.05 was taken to be statistically significant.

## 3. Results and Discussion

Nowadays, it is routinely stressed that agricultural output must be increased to keep up with the nutritional needs of the expanding global population. To this end, nanotechnology shows significant promise in many areas of agriculture, including the control of pests, pathogens, weeds, and diseases; the protection of plants; the preservation of soil and water quality; the monitoring of pollution; the improvement of crop quality; and the creation of nano-sensors [11,25].

### 3.1. Green Synthesis of Ag-NPs through Ficus Sycomorus Leaf Extract

Several approaches for NP formation have been proposed; however, the toxicity generated during production encourages green synthesis approaches as eco-friendly alternatives [26]. Currently, the use of plant extracts for NP formation has proven to be an efficient, cost-effective, and environmentally friendly approach that is widely accepted for a variety of NP preparations [27,28]. In the current study, the *F. sycomorus* leaf extract was applied as a reducing agent for the AgNO_3_ precursor for Ag-NPs formation. The Ag-NPs formation was visually indicated by the development of a dark reddish-brown color in the reduction mixture. In the same way, previous reports have shown that the color changes from green to yellow-brown [29,30,31] or dark brown [32,33,34] after the green synthesis of Ag-NPs. The ability of *F. sycomorus* leaf extract to reduce Ag^+^ ions to Ag-NPs in the current study is in line with other studies that reported Ag-, Au-, and Cu-NPs formation through *F. sycomorus* extracts [35,36,37].

### 3.2. Characterization of the Green Synthesized Ag-NPs

#### 3.2.1. Scanning Electron Microscopy (SEM) and Transmission Electron Microscopy (TEM) Analysis

**Figure 1:**
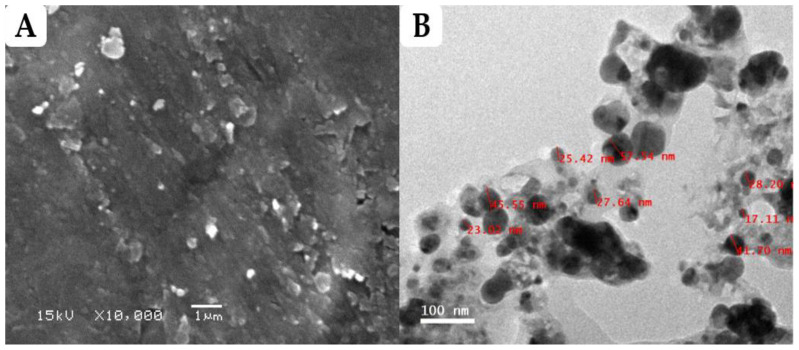
Characterization of Ag-NPs synthesized using aqueous leaf extract of *Ficus sycomorus*. (**A**) Field emission scanning electron micrograph of FS-Ag-NPs; (**B**) Field emission transmission electron micrograph of FS-Ag-NPs.

The morphological characterization of greenly prepared FS-Ag-NPs was investigated through SEM and TEM techniques. As shown in Figure 1A, the synthesized FS-Ag-NPs revealed round-shaped, compacted particles of slightly different sizes. The TEM results (Figure 1B) demonstrated the formation of nanoscale NPs with sizes ranging from 17.1 to 57.5 nm, with an average particle size of 33 ± 1 nm. Most of the particles are round shaped despite the different growth phases. The variation in the NPs’ shape and size is widely reported for green synthesized Ag-NPs and is attributed to the reduction conditions during the synthesis process [35,38]. The results in the current study align with Mishra et al., who reported nearly spherical biosynthesized Ag-NPs, according to SEM and TEM images; the size of their nanoparticles ranged from 10 to 50 nm [39]. Our findings are also in line with Salem et al. [35], who found that some of the spherically shaped and other irregularly shaped biosynthesized Ag-NPs seemed to be aggregated, according to the TEM images. Meanwhile, Spagnoletti et al. [40] reported that the organic layer of silver nanoparticles can increase particle stability by preventing aggregation. This is because the organic molecules act as a barrier between the silver nanoparticles, preventing them from coming into direct contact with each other and thus reducing the chances of aggregation. Additionally, the organic layer can also provide steric stabilization, which occurs when the organic molecules create a physical barrier around the particles, further preventing aggregation. Therefore, the presence of an organic layer can help to maintain the stability of silver nanoparticles by reducing the likelihood of aggregation. It was suggested that the smaller NPs would have a greater volume and surface area, a phenomenon that would boost their biological activity by making it easier for them to penetrate biological membranes and target cellular structures [38]. Additionally, an organic layer surrounds the Ag-NPs, as shown in Figure 1B. This organic layer may be the consequence of NPs interacting with the components of the *F. sycomorus* leaf extract phytochemicals, which is consistent with another study [41].

#### 3.2.2. Energy Dispersive X-ray Spectroscopy (EDX) and Zeta-Potential Evaluation

**Figure 2:**
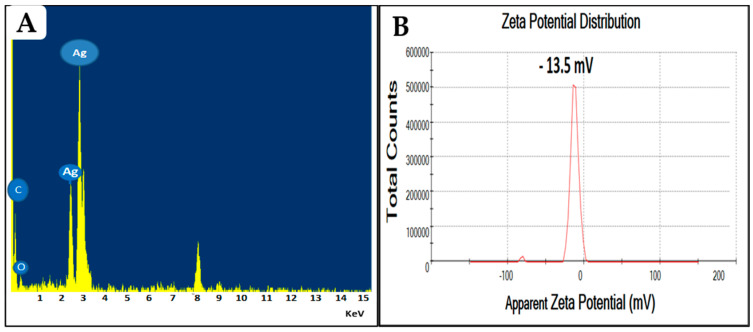
The energy dispersive X-ray spectroscopy (**A**) and zeta potential (**B**) analysis of the prepared Ag-NPs from the aqueous leaf extract of the *Ficus sycomorus* plant.

The elemental analysis of greenly prepared Ag-NPs was performed through transmission electron microscopy energy-dispersive X-ray spectroscopy (TEM-EDX) analysis. When conducting an EDX of the entire scanned area, a prominent sharp peak was observed at 3 keV, indicating the presence of metallic silver in the biosynthesized nanoparticles (Figure 2A). These results are consistent with the previous findings of Salem et al., who found that the peaks at 3 and 3.1 keV were attributed to the presence of Ag in the *F. sycomorus* [35]. The presence of two additional peaks for carbon and oxygen could be related to the organic layer surrounding the NPs; this is in line with the TEM results and with other reported studies [41,42,43]. Furthermore, the net charge of NPs is an important characteristic that directly affects both the stability and toxicity of the particles [38]. The zeta potential for the prepared FS-Ag-NPs was elucidated through Zetasizer. The obtained results revealed the net negative charge of the prepared FS-Ag-NPs surfaces with a zeta-potential of about −13.5 mV (Figure 2B). This low zeta potential suggests the good stability of the prepared Ag-NPs in liquid preparation and is attributed to strong electrostatic repulsion among NPs [28]. Furthermore, as previously reported, negatively charged NPs (Zeta-potential ≤ −30 mV) are less cytotoxic than positively charged NPs, which readily react with negatively-charged cell membranes [38,44]. This negative charge could be related to the several negatively charged functional groups (OH^−^ and COO^−^) in the FS-Ag-NPs surface, as indicated in the FTIR results.

#### 3.2.3. Fourier Transform Infrared (FTIR) Spectral Analysis

**Figure 3:**
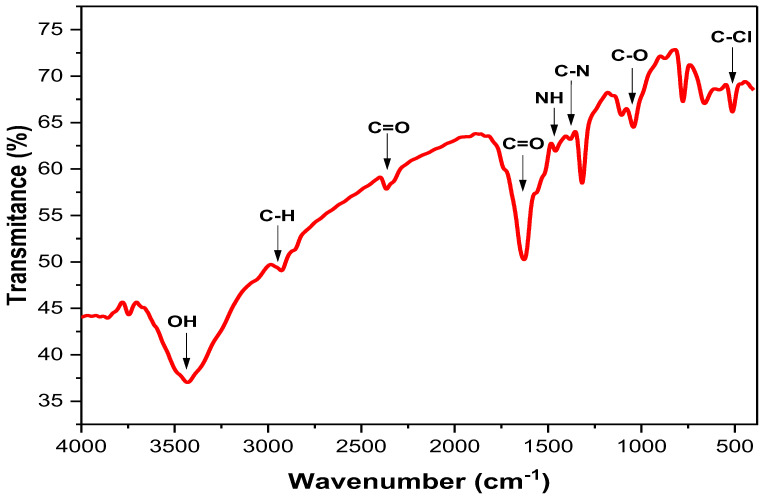
The FTIR spectra of the green synthesized Ag-NPs by *Ficus sycomorus* leaf extract.

FTIR spectroscopy was used to figure out what kinds of functional groups were in the Ag-NPs solution. Hence, it is useful for pinpointing the potential biomolecules of the plant extract involved in Ag^+^ ion reduction [12,45]. In the current study, the FTIR analysis revealed the existence of several functional groups in the prepared Ag-NPs (Figure 3). The strong-broad band detected at 3430 cm^−1^ indicated the stretching vibration of -OH (hydroxyl) group [38], while the small band at 2929 cm^−1^ indicated the stretching vibration of C-H groups [46]. Additionally, a small band was detected at 2365 cm^−1^, which confirmed the presence of C=O groups (specific to CO_2_ gas) in the prepared FS-Ag-NPs, where the strong-sharp band at 1627 cm^−1^ indicated the carbonyl groups (C=O) stretching vibration [46]. Small bands at 1382 and 1460 cm^−1^ indicated the stretching of C-N and bending of NH, respectively, which confirm the existence of aliphatic groups in amid II [38]. Other functional groups were also detected in the FTIR results, with corresponding bands at 1042 (C-O vibration), 778, 661, and 513 (C-Cl starching) cm^−1^. Hence, the FTIR analysis strongly confirmed the presence of several functional groups in the green synthesized FS-Ag-NPs, which could be attributed to organic layer coating from *F. sycomorus* leaf extract and is in line with TEM and EDX results. Raja and his colleagues attributed the stability and bioactivity of greenly synthesized Ag-NPs to the functional groups acquired from *Calliandra haematocephala* leaf extract [47].

### 3.3. Impact of Nanoparticles on the Viability/Mortality of Root-Knot Nematode

Data indicated the significant effects of FS-Ag-NPs on the viability of root-knot nematode, as illustrated in Table 1. All treatments increased the mortality percentage of the root-knot nematode by about 17.10 to 57.62% when compared with the negative control 24, 48, and 72 h post-treatment at room temperature exposure. A significant reduction was observed in the number of mortal nematodes with three different nanoparticle concentrations (50, 100, and 200 µg/mL). In addition, increasing the concentration of NPs effectively decreased the number of live nematodes.

**Figure 4:**
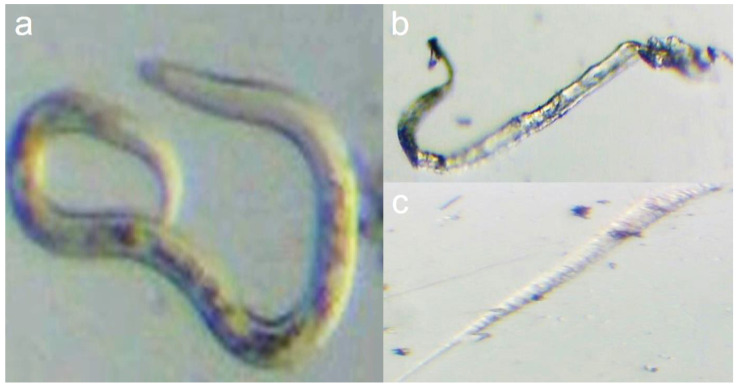
Illustrate the *Ficus sycomorus*-mediated nanoparticles effect on nematode viability. Where (**a**) shows a live control root-knot nematode, and (**b**,**c**) shows the dead nematode resulting from 200 µg/mL nanoparticles treatment.

Significant differences among different concentrations of nanoparticles were determined under a light microscope in comparison with control samples of live nematodes in Figure 4; this illustrated the impact of the nanoparticles on nematodes viability, where the highest concentration (200 µg/mL) destroyed the nematode features and also caused the highest number of dead nematodes. These encouraging findings represent the first instance of using FS-Ag-NPs synthesized from the aqueous extract of *Ficus sycomorus* as potential agents for controlling plant-pathogenic nematodes. These results are similar to those of Heflish et al. [12], where the highest concentration of their nanoparticles (100 mg/mL) caused the most increased mortality percentage of root-knot nematode. It is important to note that the effective dose of Ag-NPs required to kill J2s can vary depending on a variety of factors, including the size and stability of the nanoparticles, the type of organism being targeted, and the experimental conditions used. Therefore, it is not uncommon to see different effective doses reported in different studies. It is also possible that the differences in effective doses between studies could be due to variations in the synthesis methods and characterization of Ag-NPs, as well as the specific methods used for testing the toxicity of the nanoparticles against the J2s. Further studies are needed to better understand the factors that influence the toxicity of Ag-NPs against nematodes and other organisms.

The concentration of FS-Ag-NPs needed to kill the J2s was found to be higher in our study, as other studies have reported the use of higher concentrations ranging from 20 to 150 ppm [48,49] and 125–1000 mg/L [50]. Our findings are not in agreement with previous reports by Baronia et al. [51], who achieved 100% mortality at a concentration of 2 ppm after 24 h of Ag-NPs treatment, and Rani et al. [11], who observed complete death of *Meloidogyne incognita* J2s at a low dose of 6 ppm after 12 h of treatment with *Glycyrrhiza glabra*-silver nanoparticles synthesized.

### 3.4. Effect of FS-Ag-NPs Exposure on the Studied Bacterial Isolates

The Ag-NPs produced from *Ficus sycomorus* demonstrated the most significant inhibition zones against *Ralstonia solanacearum*, with average values ranging from 14.00 ± 2.16 to 26.00 ± 2.83 mm. This is compared to the positive control (amoxicillin antibiotic) value of 16.33 ± 0.94 mm. The values are more significant than those reported using Ag-NPs obtained from Fusarium genera against human pathogens such as *E. coli* and *S. aureus* [52,53]. The antibiotic amoxicillin showed the highest zone of inhibition, with an average of 19.33 ± 0.47 mm against *Pectobacterium atrosepticum*. The negative control did not exhibit growth inhibition, as shown in Table 2.

**Figure 5:**
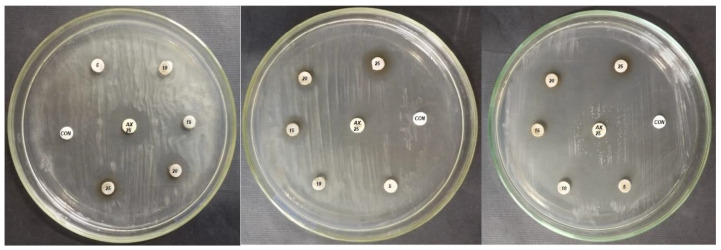
*Ficus sycomorus*-mediated nanoparticles’ effect on different bacterial isolates. The discs marked with CON = negative control (sterile double distilled water); AX 25 = amoxicillin 25 µg (positive control); the discs show concentrations of 5, 10, 15, 20, and 25 µg/mL of FS-Ag-NPs.

The study’s findings indicate that the produced Ag-NPs exhibit antibacterial activity against the phytopathogenic agent *P. carotovorum*, with average values ranging from 10.33 ± 0.94 to 15.67 ± 0.94 mm. However, the isolate *P. atrosepticum* showed the lowest activity, with inhibition halos ranging from 9.67 ± 0.94 to 15.33 ± 0.47 mm, as shown in Table 2. Guzman et al. [54] previously reported that Ag-NPs exhibited antibacterial activity against Gram-negative bacteria. The visible clear zones produced by FS-Ag-NPs against three different Gram-negative bacteria are shown in Figure 5.

The FS-Ag-NPs produced in this study were found to be smaller in size (33 nm) and highly effective against bacteria, which is consistent with previous research indicating that smaller nanoparticles have a larger surface area and greater susceptibility to antibacterial agents [55]. In addition to size, it is important to consider other factors, such as biocompatibility and stabilization by organic compounds, when synthesizing Ag-NPs through biosynthesis. Silver nanoparticles are known to be effective against drug-resistant bacteria due to their unique chemical and physical properties, with particle sizes typically ranging from 1 to 100 nm. Research has shown that smaller nanoparticles have a greater surface area-to-volume ratio, which enhances their ability to adhere to and penetrate bacteria cells, thereby improving their antimicrobial activity [56].

The antibacterial properties of FS-Ag-NPs against *R. solanacearum* and *P. carotovorum* can be attributed to their multiple mechanisms of action. These mechanisms can act on both the cell wall and membrane, which can lead to structural changes in the cell. Additionally, FS-Ag-NPs have been shown to affect the functionality of proteins and DNA, which are essential to various cell processes, including electron transport in the respiratory chain, protein synthesis, cell permeability, and replication of genetic material [52,53]. These findings demonstrate the potential of FS-Ag-NPs as an effective control agent for managing soft rot disease and provide a basis for implementing this approach on a larger scale.

Silver nanoparticles have been recognized for their broad-spectrum antimicrobial properties, which have been used for the prevention and treatment of various diseases. They have demonstrated efficacy as antifungal, anti-inflammatory, and antiviral agents [57,58,59]. With the advent of non-toxic synthesis methods, cost-effective Ag-NPs can be produced and tested as new antimicrobial agents. This study evaluated the use of Ag-NPs as an antimicrobial agent against selected Gram-negative bacteria in an agar plate. The results indicated complete inhibition of bacterial growth, with the degree of inhibition dependent on the concentration of Ag-NPs and the type of bacteria used in the experiments [56]. Ag-NPs have been shown to have a distinct advantage over conventional chemical antimicrobial agents, especially in agriculture, due to antibiotic resistance issues. Traditional chemical antimicrobial agents rely on specific binding between microorganisms and the surface and metabolites of the antimicrobial agents, which can lead to the development of multiple resistance traits in various organisms over generations. In contrast, bacteria are less likely to develop resistance to metal nanoparticles such as Ag-NPs, making them a potential alternative to conventional antibiotics for overcoming antibiotic-resistant microorganisms.

## 4. Conclusions

This study synthesized silver nanoparticles using *Ficus sycomorus* leaves (FS-Ag-NPs) and tested their effectiveness against plant pathogens. The nanoparticles were characterized using various methods, and the results showed that the synthesized NPs were nanoscale. We found that the FS-Ag-NPs were effective against the root-knot nematode and several bacterial strains. According to the research, the utilization of FS-Ag-NPs could be a suitable solution for controlling plant-parasitic nematodes because of their ease of use, stability, affordability, and environmentally friendly characteristics.

## Figures and Tables

**Table 1:** The efficiency of the synthesized *Ficus sycomorus* silver nanoparticle (FS-Ag-NPs) concentrations on root-knot nematode after exposure to consequence intervals.

Number of Live Nematodes		Number of Dead Nematodes after Treatment of FS-NPs ± SD * (µg/mL)						Exposure Time Intervals
		50	M% **	100	M%	200	M%	
Control 1	95.33	40 ±6.53	21.68	38.33 ± 1.70	40.21	20.67 ± 3.09	41.96	24 h
Control 2	89.67	51.67 ± 5.31	17.10	20 ± 1.63	22.30	15.33 ± 3.40	57.62	48 h
Control 3	129	67 ± 5.35	28.68	25.67 ± 2.87	19.90	37± 2.16	51.94	72 h

* SD = standard deviation, ** M% = mortality percentage.

**Table 2:** Response of different bacterial isolates to the synthesized *Ficus sycomorus* silver nanoparticles (FS-Ag-NPs) recorded as inhibition zone (mm) using the disc diffusion method.

Concentration (µg/mL)	Bacterial Isolates Inhibition Zone (mm ± SD)		
	*Pectobacterium carotovorum* (MN598002)	*P. atrosepticum* (MG706146)	*Ralstonia solanacearum* (LN681200)
5	10.33 ± 0.94 c	9.67 ± 0.94 d	14.00 ± 2.16 c
10	10.00 ± 0.82 c	9.33 ± 0.47 d	17.33 ± 2.05 c
15	12.00 ± 1.41 c	10.67 ± 0.94 c	19.00 ± 1.41 bc
20	15.33 ± 0.47 b	12.67 ± 1.25 cd	24.00 ± 1.41 ab
25	15.67 ± 0.94 b	15.33 ± 0.47 b	26.00 ± 2.83 a
NC *	0.00 ± 0 d	0.00 ± 0 e	0.00 ± 0 d
PC **	19.67 ± 1.7 a	19.33 ± 0.47 a	16.33 ± 0.94 c

* NC = negative control (sterile double distilled water); ** PC = positive control (Amoxicillin 25 µg). If the articles next to the data in each column are different, it means that the data were significantly different with a probability of 0.01.

## Data Availability

Experimental data supporting the findings of this study are available from the corresponding authors (A.A. and S.B.) upon request.
